# The Frequency of Intestinal Parasitic Infections in COVID-19 Patients: A Case-Control Study in Tehran, Capital of Iran

**DOI:** 10.1155/2023/5359823

**Published:** 2023-09-08

**Authors:** Ali Taghipour, Majid Pirestani, Ramin Hamidi Farahani, Mohammad Barati

**Affiliations:** ^1^Zoonoses Research Center, Jahrom University of Medical Sciences, Jahrom, Iran; ^2^Department of Parasitology, Faculty of Medical Sciences, Tarbiat Modares University, Tehran, Iran; ^3^Infectious Diseases Research Center, AJA University of Medical Sciences, Tehran, Iran

## Abstract

The present study was done to evaluate the prevalence of intestinal parasitic infections (IPIs) in patients with COVID-19 in health care centers (Imam Reza and Golestan hospitals), Tehran, capital of Iran. By designing a matched case-control study, 200 fecal samples were collected for each of the COVID-19 patients and healthy individuals. Nasopharyngeal/oropharyngeal swab samples were collected from all participants for the diagnosis of COVID-19. RNA extraction was performed, and then real time reverse-transcription polymerase chain reaction (rRT-PCR) assay was applied to detect viral RNA. Considering the lung complications, 25%> lung complications was detected in 49 patients, 25–49% in 42 patients, and 50%≤ in 109 patients. Fecal samples were examined using different parasitological techniques. After nested-PCR, sequencing was applied to identify *Cryptosporidium* spp. and microsporidia spp. A relatively lower prevalence of IPIs was detected among control group (7.5%), than in COVID-19 patients (13%), though not significant (*P*=0.13). The most prevalent parasite among patients was *Blastocystis* sp. (6%). Also, 13.76% of IPIs were detected in inpatients with more than 50% lung complication. As well, a remarkably significant difference in IPIs was observed among diarrheic COVID-19 patients, in comparison with nondiarrheic patients (*P* < 0.00001). Moreover, the isolated sequences in the present study belonged to *C. parvum* subtype IIa and *Enterocytozoon bieneusi* genotypes D and Peru 8. In conclusion, more epidemiological and clinical research studies are needed to better understand the status and interaction of IPI in COVID-19 in Iran and other countries.

## 1. Introduction

A strain of Coronavirus called severe acute respiratory syndrome coronavirus 2 (SARS-CoV-2) was first emerged at the end of December 2019 in Republic of China (Wuhan City of Hubei Province) and quickly spread around the world [1–3]. Although manifestations of COVID-19 are typically asymptomatic or mild in immunocompetent individuals, endothelial damage and acute respiratory distress syndrome (ARDS) are the main concerns in patients with underlying diseases [3–6]. The case fatality rate due to COVID-19 was reported 1% in general population, 13% in hospitalized patients, 19% in patients older than 50 years, and 37% in patients admitted to intensive care unit (ICU) [7]. During a meta-analysis study, 19% of coinfections and 24% of super-infections were reported in patients with COVID-19 [8]. Increased mortality is one of the most important consequences of these coinfections or super-infections.

About 3.5 billion people, mostly in developing countries, are affected by the intestinal parasitic infections (IPIs) worldwide [9, 10]; in this sense, *Ascaris lumbricoides*, *Trichuris trichiura*, and hookworms, most prevalent soil-transmitted helminths (STHs), affect about 447, 290, and 229 million individuals, respectively [11]. Additionally, intestinal protozoan parasites are less frequent than STHs, with 184 million (*Giardia lamblia*), 104 million (*Entamoeba histolytica*), and 64 million (*Cryptosporidium* spp.) patients, respectively [12]. The IPIs can seriously impact not only the digestive functions of the affected host but also the immunological balance of the body [13–15]. Potentially, STHs and intestinal protozoa can stimulate the T helper 2 (Th2) and T helper 1 (Th1) cells, respectively [13–17]. On the other hand, coinfections of IPIs and some intracellular pathogens, namely, *Mycobacterium tuberculosis* and human immunodeficiency virus (HIV), may cause an imbalance in the host and more pathological complications [13, 14, 16, 17]. Since the emergence of the COVID-19, there have been some theories on the possible interaction between the IPIs and COVID-19 [18, 19]. Thus, the present case-control study was done to survey the frequency of the IPIs among COVID-19 patients and healthy individuals.

## 2. Materials and Methods

### 2.1. Study Population

The fecal samples were collected from the health centers of Tehran, capital of Iran (Imam Reza and Golestan hospitals) from April 2021 to May 2022. Confirmation of COVID-19 and consent to participate were inclusion criteria, and presence of immunodeficiency and use of anti-parasitic drugs during the last three months prior to sampling were exclusion criteria. Before collecting fecal samples, a written informed consent was obtained from all subjects. In accordance with the WHO recommendations regarding the diagnosis of COVID-19, nasopharyngeal/oropharyngeal swab samples were collected from all participants for the diagnosis of COVID-19. RNA extraction was performed by Viral Nucleic Acid Kit, and then real time reverse-transcription polymerase chain reaction (rRT-PCR) assay was applied to detect viral RNA [20]. In the following, 200 fecal samples were collected for each of the COVID-19 patients and control group. Non-COVID-19 individuals (negative for COVID-19 test) and without any history of COVID-19 were confirmed by the physician as the control group. Age, gender, and place of residence were matched between both case-control groups in order to improve the accuracy of the study. A questionnaire including sociodemographic features and clinical symptoms related to IPI was filled by each participant.

### 2.2. Identification of IPIs

For the detection of trophozoite and cyst stages of protozoa, direct smears (normal saline and Lugol's iodine staining) were used in accordance with the available standard protocols. Also, fecal concentration by formalin-ether with Lugol's iodine staining was applied for protozoan cysts. At the identification level, trichrome staining was used to detect *G. lamblia* and *E. histolytica*, modified Ziehl–Neelsen was employed to identify the *Cryptosporidium* spp. oocysts, and a chromotrope 2R staining was done for the microsporidian agents [21]. After usual formalin-ether concentration, iodine staining was applied for the detection of helminths ova and larvae.

The slides related to the samples were examined by using the light microscope (Zeiss, Germany), under 10×, 40×, and 100× magnification along with the positive control. Regarding DNA extraction for *Cryptosporidium* spp. and microsporidia, part of the fecal samples was stored in 70% alcohol at 4°C.

### 2.3. Molecular Examination

The molecular examination of the positive and some negative specimens (negative specimens that patients had gastrointestinal disorders) was done regarding *Cryptosporidium* spp. and microsporidia. The genomic DNA was extracted from 200 mg of the fecal sample using a DNA purification kit (Yekta Tajhiz Azma Co., Iran), based on the manufacturer's instructions. A nested-PCR assay was done using primer pairs and assay conditions described previously [21]. After electrophoresis in 1.5% agarose gel, PCR products were revealed by ultraviolet light. By Applied Biosystems 3730/3730xl DNA Analyzers (Bioneer, Korea), PCR products of positive samples were sequenced and the results of our study were compared with samples available in GenBank by BLAST software.

### 2.4. Statistical Analysis

For the data analysis, the Chi-square and Fisher's exact tests were applied for variables of cases and controls using SPSS software version 16 (SPSS, Chicago, IL, USA). *P* values less than 0.05 were indicated statistically significant.

## 3. Results

**Figure 1:**
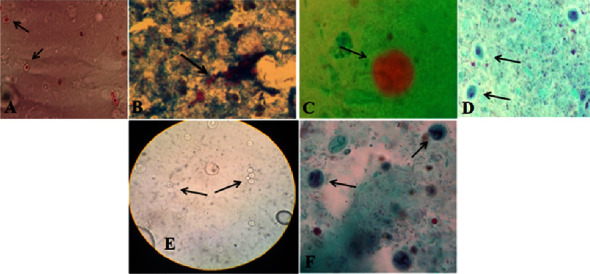
Identified intestinal protozoa among the COVID-19 group in the present study. (A) Microsporidia spp., (B) *Cryptosporidium* spp., (C) *Entamoeba coli,* (D) *Chilomastix mesnili,* (E) *Blastocystis* sp., and (F) *Giardia lamblia*.

A total of 400 subjects were included in this study. Among the included subjects, 200 COVID-19 patients (53.5% male and mean age of 47.14 ± 12.29 years) and 200 individuals without COVID-19 (51% male and mean age 47.77 ± 11.57 years) were confirmed [22]. Considering the lung complications, 25%> lung complications was detected in 49 patients, 25–49% in 42 patients, and 50%≤ in 109 patients. The frequency of IPIs in patients with COVID-19 (13%; 26/200) was higher than that in individuals without COVID-19 (7.5%; 15/200) although no statistically significant difference was found (*P* value = 0.13). Infection with intestinal helminths was not detected in both groups. Identified intestinal protozoa in the COVID-19 group were *Blastocystis* sp., *G. lamblia*, *Entamoeba coli*, *Chilomastix mesnili*, microsporidia spp., and *Cryptosporidium* spp (Figure 1). The frequencies of different IPIs in the groups with COVID-19 and without COVID-19 are presented in Table 1. Regarding the associated factors for IPIs, we did not find significant differences regarding the age, gender, residence, and duration of treatment among parasitized and nonparasitized people in the patients with COVID-19 (Table 2). Also, 13.76% of IPIs were detected in inpatients with more than 50% lung complication (Table 2). However, a statistically significant difference was seen for the frequency of IPIs among diarrhea patients compared to nondiarrhea patients in the COVID-19 patients (*P* value <0.00001) (Table 2). In this regard, a statistically significant difference was observed between the frequency of IPIs in patients with COVID-19 who had loose and watery stools (*P* value <0.00001) (Table 2).

**Figure 2:**
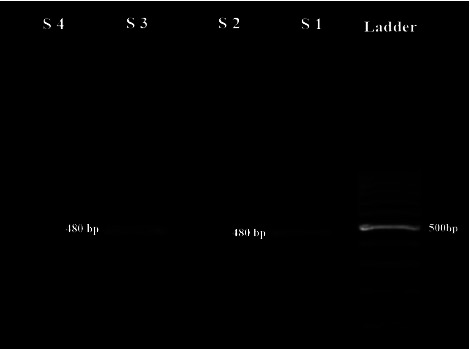
Identification of *Cryptosporidium parvum* using nested-PCR. Gel electrophoresis of 480 bp fragment of gp60 gene of *C. parvum*; S1: positive control, S2 and S4: negative control, and S3: positive sample of this study.

**Figure 3:**
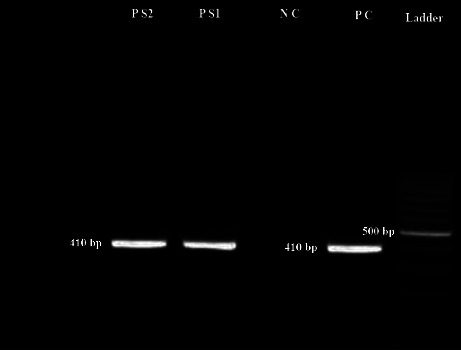
Identification of *Enterocytozoon bieneusi* using nested-PCR. Gel electrophoresis of 410 bp fragment of ssu rRNA gene of *E. bieneusi*; PC: positive control, NC: negative control, and PS1 and PS2: positive samples of this study.

As shown in Table 1, two cases of microsporidia spp. (1%) and one case of *Cryptosporidium* spp. (0.5%) were detected in patients with COVID-19 and the frequency of these opportunistic protozoa was not significantly different in the COVID-19 patient and healthy groups (*P* value = 0.55 for microsporidia spp. and *P* value = 0.54 for *Cryptosporidium* spp.). Molecular examinations revealed expected fragments for *Cryptosporidium* spp. and *Enterocytozoon bieneusi* (Figures 2 and 3). The results of PCR on 50 randomly selected *Cryptosporidium* spp. and microsporidia-negative fecal samples showed negative amplification. Only microscopically positive samples were positive by PCR. Sequencing and BLAST indicated a positive isolate for *Cryptosporidium parvum* with subtype IIa. For *E. bieneusi*, genotypes D and Peru 8 were identified. We deposited the sequences we identified for *E. bieneusi* (accession no. ON682481 and ON682482) and *C. parvum* (accession no. ON932599) in GenBank.

With respect to the ethical concerns, parasitological data obtained for each patient with COVID-19 followed by choice drugs recommendation regarding IPIs were dispatched to the specialist clinicians for further clinical evaluation and practice.

## 4. Discussion

The present findings revealed that 13% of COVID-19 patients were infected with intestinal protozoa. These results are much lower than the frequency of IPIs from tuberculosis (TB) patients (21.1%) [21], hemodialysis patients (28.4%) [23], and HIV patients (48.8%) [24] among Iranian population. In general, it seems that the prevalence of IPIs has reduced during the COVID-19 pandemic in Iran [25]. However, Teimouri et al. showed that the prevalence of IPIs was higher among that referred to hospitals before the COVID-19 pandemic (5.8%) than during the COVID-19 pandemic (2.8%), with a statistically significant difference (*P* value <0.001) [25]. In the present study, the prevalence of IPIs was higher in patients with COVID-19 than in the healthy group, although statistical significance was not observed (*P* value = 0.13), which can be explained related to increasing attention and improving attitudes to personal and social hygiene and health-related behaviors during the COVID-19 pandemic. In this regard, the results of our study showed that no helminthic infection was found in both groups. An epidemiological study in Ethiopia reported that 37.81% (284 of 751) COVID-19 patients were infected with intestinal parasites [26]; this result was three times the prevalence of our study. Several parameters may contribute to the dispersion of IPIs, including high or low Human Development Index (HDI), geographic region, and demographic characteristics [27–29].

Our results demonstrated the predominance of the intestinal protozoan, *Blastocystis*, in COVID-19 patients, being consistent with some recent studies on various populations in Iran [21, 25, 30]. The potential role of *Blastocystis* sp. in pathogenic is debatable, since researchers have revealed its contribution in gastrointestinal manifestations, while some researchers have rejected this association [31–33]. In the present study, three types of pathogenic protozoa (six cases of *G. lamblia*, two cases of microsporidia, and one case of *Cryptosporidium* spp.) were found.

Considering the molecular survey, it was verified that two isolates of microsporidia spp. were *E. bieneusi* and one isolate of *Cryptosporidium* spp. was *C. parvum*, which is in line with previous studies from Iran among TB patients [21] and immunocompromised individuals [34, 35]. Moreover, *E. bieneusi* genotypes recognized in this study included D and Peru 8, in which genotype D is the most common in Iran [21, 34].

Considering the risk factors, we found that diarrhea patients with COVID-19 were more likely to be infected with intestinal parasites, which may be attributed to the high prevalence of IPIs in these participants. However, diarrhea cannot be completely attributed to IPIs because some infectious and/or non-infectious agents may contribute to diarrhea. Also, a meta-analysis study has shown that COVID-19 appears to be more serious in patients with gastrointestinal symptoms [36]. The severe rate of COVID-19 patients with diarrhea was 41.1%, and the odds ratio of association between diarrhea and severe COVID-19 was 1.41 (95% CI: 1.05–1.89) [36]. Therefore, the relationship between IPIs and diarrhea cannot be reliably predicted and requires extensive future research.

As a final word, about 13% of COVID-19 patients were infected with IPIs, in comparison with 7.5% in healthy individuals. In both groups, *Blastocystis* sp. was the most prevalent parasitic agent. Health education along with observation of personal and social hygiene is highly recommended to prevent IPIs in COVID-19 patients. Moreover, more epidemiological and clinical research studies are needed to better understand the status and interaction of IPI in COVID-19 in Iran and other countries.

## Figures and Tables

**Table 1:** Frequency of intestinal parasitic infections among COVID-19 patients and healthy group.

Parasites	COVID-19 patients (*n* = 200)	Healthy group (*n* = 200)	
	No. of positive (%)	No. of positive (%)	*P* value
*Blastocystis* sp.	12 (6%)	8 (4%)	0.48
*Giardia lamblia*	3 (1.5%)	3 (1.5%)	1
*Entamoeba coli*	3 (1.5%)	2 (1%)	0.65
*Chilomastix mesnili*	2 (1%)	2 (1%)	1
Microsporidia spp.	2 (1%)	0 (0%)	0.55
*Cryptosporidium* spp.	1 (0.5%)	0 (0%)	0.54
*G. lamblia* + *Entamoeba coli*	2 (1%)	0 (0%)	0.55
*Blastocystis* sp. *+* *G. lamblia*	1 (0.5%)	0 (0%)	0.54

*P* values <0.05 are statistically significant.

**Table 2:** Age, gender, type of stool, diarrhea status, residence, type of patients, lung complications, and duration of treatment of patients with COVID-19 and healthy group, according to the presence or absence of intestinal parasites.

Variables	COVID-19 patients (*n* = 200)				Healthy group (*n* = 200)			
	No. of examined	No. of positive (%)	No. of negative (%)	*P* value	No. of examined	No. of positive (%)	No. of negative (%)	*P* value
*Age group (year)*								
20–30	20 (10%)	1 (5%)	19 (95%)	0.65	18 (9%)	0 (0%)	18 (100%)	0.71
31–40	44 (22%)	5 (11.37%)	39 (88.63%)		44 (22%)	4 (9.09%)	40 (90.91%)	
41–50	52 (26%)	6 (11.54%)	46 (88.46%)		48 (24%)	6 (12.5%)	42 (87.5%)	
51–60	51 (25.5%)	10 (19.60%)	41 (80.40%)		64 (32%)	4 (6.25%)	60 (93.75%)	
61–70	33 (16.5%)	4 (12.12%)	29 (87.88%)		26 (13%)	1 (3.84%)	25 (96.16%)	

*Sex*								
Male	107 (53.5%)	16 (14.95%)	91 (85.05%)	0.53	102 (51%)	9 (8.82%)	93 (91.18%)	0.50
Female	93 (46.5%)	10 (10.75%)	83 (89.25%)		98 (49%)	6 (6.12%)	92 (93.88%)	

*Residence*								
Urban	151 (75.5%)	16 (10.60%)	135 (89.40%)	0.21	145 (72.5%)	10 (6.90%)	135 (93.10%)	0.62
Rural	49 (24.5%)	10 (20.40%)	39 (79.60%)		55 (27.5%)	5 (9.10%)	50 (90.90%)	

*Type of stool*								
Formed	61 (30.5%)	1 (1.64%)	60 (98.36%)	<0.00001^*∗*^	71 (35.5%)	4 (5.63%)	67 (94.37%)	0.87
Soft	101 (50.5%)	9 (8.91%)	92 (91.09%)		99 (49.5%)	9 (9.09%)	90 (90.91%)	
Loose	18 (9%)	7 (38.88%)	11 (61.12%)		17 (8.5%)	1 (5.88%)	16 (94.12%)	
Watery	20 (10%)	9 (45%)	11 (55%)		13 (6.5%)	1 (7.69%)	12 (92.31%)	

*Diarrhea*								
Yes	38 (19%)	16 (42.10%)	22 (57.90%)	<0.00001^*∗*^	30 (15%)	2 (6.66%)	28 (93.34%)	0.86
No	162 (81%)	10 (6.17%)	152 (93.83%)		170 (85%)	13 (7.64%)	157 (92.36%)	

*Duration of treatment of patients with COVID-19*								
2 weeks	61 (30.5%)	4 (6.55%)	57 (93.45%)	0.10	—	—	—	—
2–4 weeks	43 (21.5%)	4 (9.30%)	39 (90.70%)		—	—	—	—
4–6 weeks	25 (12.5%)	3 (12%)	22 (88%)		—	—	—	—
6 weeks<	71 (35.5%)	15 (21.12%)	56 (78.88%)		—	—	—	—

*Type of patients*								
Inpatients	109 (54.5%)	15 (13.76%)	94 (86.24%)	0.55	—	—	—	—
Outpatients	91 (45.5%)	11 (12.09%)	80 (87.91%)		—	—	—	—

*Lung complications*								
25%>	49 (24.5%)	6 (12.24%)	43 (87.76%)	0.77	—	—	—	—
25–49%	42 (21%)	5 (11.90%)	37 (88.10%)		—	—	—	—
50%≤	109 (54.5%)	15 (13.76%)	94 (86.24%)		—	—	—	—

*P* values <0.05 are statistically significant.

## Data Availability

All data used during study are included in this manuscript.
